# Using linked educational attainment data to reduce bias due to missing outcome data in estimates of the association between the duration of breastfeeding and IQ at 15 years

**DOI:** 10.1093/ije/dyv035

**Published:** 2015-04-08

**Authors:** Rosie P Cornish, Kate Tilling, Andy Boyd, Amy Davies, John Macleod

**Affiliations:** School of Social and Community Medicine, University of Bristol, Bristol, UK

**Keywords:** Breastfeeding, IQ, linkage, missing data, administrative data, bias, ALSPAC

## Abstract

**Background:** Most epidemiological studies have missing information, leading to reduced power and potential bias. Estimates of exposure-outcome associations will generally be biased if the outcome variable is missing not at random (MNAR). Linkage to administrative data containing a proxy for the missing study outcome allows assessment of whether this outcome is MNAR and the evaluation of bias. We examined this in relation to the association between infant breastfeeding and IQ at 15 years, where a proxy for IQ was available through linkage to school attainment data.

**Methods:** Subjects were those who enrolled in the Avon Longitudinal Study of Parents and Children in 1990–91 (*n* = 13 795), of whom 5023 had IQ measured at age 15. For those with missing IQ, 7030 (79%) had information on educational attainment at age 16 obtained through linkage to the National Pupil Database. The association between duration of breastfeeding and IQ was estimated using a complete case analysis, multiple imputation and inverse probability-of-missingness weighting; these estimates were then compared with those derived from analyses informed by the linkage.

**Results:** IQ at 15 was MNAR—individuals with higher attainment were less likely to have missing IQ data, even after adjusting for socio-demographic factors. All the approaches underestimated the association between breastfeeding and IQ compared with analyses informed by linkage.

**Conclusions:** Linkage to administrative data containing a proxy for the outcome variable allows the MNAR assumption to be tested and more efficient analyses to be performed. Under certain circumstances, this may produce unbiased results.

**Key Messages**

- IQ measured in ALSPAC at 15 years is missing not at random, conditional on a number of baseline socio-demographic factors.
- Incorporating a proxy for a study outcome, obtained via linkage to administrative or routine datasets:
  ○ allows some assessment of whether the study outcome is missing not at random
  ○ may enable more accurate prediction of the missing values
  ○ can potentially reduce bias in estimated exposure-outcome associations.

## Introduction

A key strength of longitudinal studies is their ability to investigate causality by making a temporal distinction between exposures and outcomes. However, they typically face problems of missing data arising through attrition or non-response. A variable is missing not at random (MNAR) if the probability of it being missing is related to its own (unknown) value, even after taking account of observed factors that predict non-response, and missing at random (MAR) if the probability of it being missing only depends on observed data. If an outcome variable is MNAR then (in most circumstances) both a complete case analysis, in which only subjects with complete information on all variables needed for a particular analysis are included, and a standard implementation of multiple imputation (MI), which assumes data are missing at random (MAR), will be biased.^1^ In contrast, if the outcome is MAR both will produce unbiased results.^1^ Unfortunately, it is not usually possible to tell whether a variable is MAR or MNAR. Instead, sensitivity analyses are typically undertaken in order to ascertain the impact of particular assumptions on the results. Linkage to administrative or other external datasets provides a means of obtaining a proxy for the study outcome, which could help determine whether this outcome is MAR or MNAR; this proxy could also be incorporated into statistical analyses.

Robust evidence suggests that breastfeeding is associated with higher childhood and adolescent cognition,^2–5^ although this association could be due to confounding by parental cognitive outcomes, socioeconomic factors or differences in parenting behaviour and interactions between mother and infant.^2^^,^^6^^,^^7^ In the Avon Longitudinal Study of Parents and Children (ALSPAC), the mean IQ at age 8 among those breastfed for 6 months or longer was 3 points higher than among those breastfed for less than 1 month or never.^8^ Here we use linked educational attainment data to model the mechanism for missing IQ in order to investigate whether this association, seen earlier in ALSPAC, extends into adolescence. Different approaches are used to take account of missing IQ, and results obtained from analyses incorporating linked attainment data are compared with those obtained from models excluding attainment, in order to evaluate the extent of bias in the latter. It is important to note that we do not claim to have fully adjusted for confounders; rather, the focus is on a comparison of the approaches and the sensitivity of the conclusions to these.

## Methods

### Subjects

ALSPAC is a prospective study of children due to be born between 1 April 1991 and 31 December 1992 to women living in and around Bristol, a city in the south-west of England.^9^ In the initial recruitment phase 14 541 pregnant women enrolled, resulting in 14 062 live births (13 988 alive at 1 year). This study uses data from all singletons and twins (*n* = 13 975). Detailed data were collected during the pregnancies and participants have been followed up since birth through questionnaires, clinics and linkage to routine datasets. (ALSPAC has a searchable data dictionary, [http://www.bris.ac.uk/alspac/researchers/data-access/data-dictionary/], describing all available data.) Ethical approval was obtained from the ALSPAC Ethics and Law Committee and local research ethics committees. Attrition rates in ALSPAC were highest in infancy and late adolescence^9^ and previous analyses have shown that those who continue to participate are more likely to be female and White and less likely to live in low income households.^9^

### Linkage between ALSPAC and the National Pupil Database

The National Pupil Database (NPD) is a longitudinal database containing attainment and other data for children attending schools in England at [https://www.gov.uk/government/collections/national-pupil-database]. Linkage between ALSPAC and the NPD was conducted in 2002 by the Fischer Trust (an independent charity) as a trusted third party. The linkage was deterministic, using name, date of birth, sex and address. Contribution to the NPD is compulsory for schools that follow the national curriculum, although provision of data to researchers is subject to consent. Individuals who attended an independent (fee-paying) school at the time of the linkage would not have been linked unless their school contributed data voluntarily, since these schools do not necessarily follow the national curriculum. Conversely, attainment data for those who were in a state school at the time of linkage but subsequently attended an independent school will be present as long as they sat GCSEs (General Certificates of Secondary Education).

### ALSPAC data

IQ was measured at 15 years during a study clinic using the Wechsler Abbreviated Scale of Intelligence (WASI).^10^ Just under 10 000 individuals were invited to this clinic, of whom around 50% attended. Most of the uninvited individuals had either died, withdrew from the study (or specifically from attending study clinics) or were untraceable. For practical reasons, only two of the four WASI subtests (Vocabulary and Matrix Reasoning) were administered; together, these provide a measure of general cognitive functioning.

The breastfeeding information used here was collected via questionnaires administered at 4 weeks, 6 months and 15 months. Duration of breastfeeding was categorized, with those who reported breastfeeding for less than 1 month combined with those who never breastfed.

A number of factors thought to be potential confounders and/or predictive of non-response were collected during pregnancy and early infancy: the child’s sex and ethnicity; smoking during the first trimester of pregnancy; maternal age and parity; mother’s and father’s educational level (classified as O level or lower, A level and degree or higher); mother’s and father’s occupational social class; housing tenure; and family adversity index, a composite measure of social adversity taking into account a variety of risk factors thought to be important for mental health outcomes (including items relating to housing, financial difficulties, family size and problems, maternal age and education, substance abuse and crime^11^). Family occupational social class was defined as the higher of maternal and paternal social class and categorized as I-IIIN (professional, managerial and non-manual skilled occupations) and IIIM-IV (manual skilled, semi-skilled and unskilled occupations).

### Linked education data

Two variables were used from the NPD, both from Key Stage 4 attainment data. Key Stage 4 refers to the 2 school years attended between 14 and 16 years. In this period, pupils typically take GCSE or equivalent vocational courses in a number of subjects and then receive a grade in each. Each grade is equivalent to a specified number of points, higher scores indicating higher attainment. The first variable used in this study was the number of A*–C grades obtained, dichotomized as <5 or 5 or more. The other variable, the capped point score (hereafter referred to as attainment score), is the total score of an individual’s top eight qualifications ranked in terms of points. The number of GCSE qualifications taken by a pupil varies, but the majority take at least eight; the capped score therefore provides a more standardized measure of attainment than an individual’s total score.

### Statistical methods

We used logistic regression to determine the predictors of missing IQ; factors included were all the baseline factors described above, including breastfeeding, and both attainment variables. We then investigated the association between attainment score and IQ. Since this relationship appeared to be non-linear, we used fractional polynomials to select the most appropriate model.^12^ The best fitting one-power fractional polynomial predicted IQ from attainment score cubed. Linear regression was used to model the relationship between duration of breastfeeding and IQ. We used five approaches to dealing with missing IQ data: a complete case analysis; inverse probability-of-missingness weighting (IPMW),^13^ both including and excluding the attainment data; and multiple imputation using chained equations (fully conditional specification), also performed both with and without attainment data. As described above, both logistic models used to obtain the inverse probability weights included all baseline variables; the second model also included both attainment variables. The Hosmer–Lemeshow goodness of fit test^14^ was used to assess the fit of these. As a sensitivity analysis, large weights were truncated—choosing different maximum values (8, 6 and 4). The MI models included all baseline variables, as these were all thought a priori to be confounders of the breastfeeding-IQ association, predictive of missing IQ, or both. In addition to these, in the MI models incorporating attainment data IQ was imputed from attainment score cubed and attainment score from the cube root of IQ; the equations for all other variables included attainment score as a linear variable. The dichotomous attainment variable was also included in these MI models. Further, because it was hypothesised that the relationship between attainment and IQ might differ by socio-economic status, the imputation models for IQ and attainment score also included an interaction between mother’s education and attainment score cubed/the cube root of IQ (respectively). For each analysis, 100 datasets were imputed. Finally, as a sensitivity analysis we deducted a fixed number of points from the imputed IQs of all individuals with missing attainment data (i.e. individuals with missing IQ and attainment) – under the assumption that their imputed IQ would be an overestimate because children who attend independent schools would, on average, obtain better GCSE grades for a given IQ. Analyses were carried out using Stata 13.0; multiple imputation used the mi impute command.

## Results

**Figure 1.**
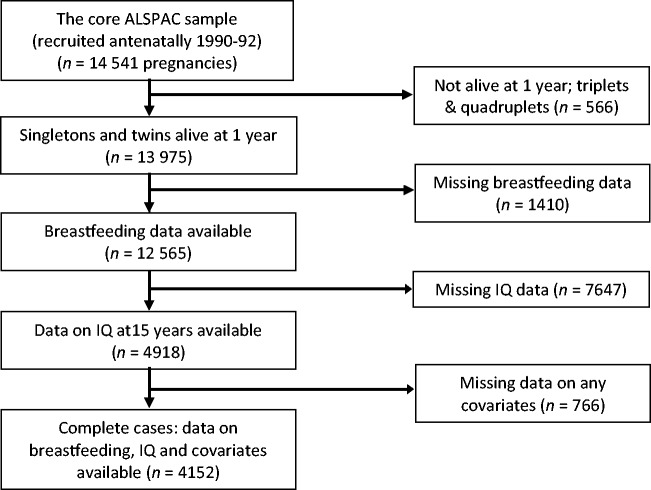
Flowchart showing ALSPAC data available for this study.

Of the 13 975 subjects included in this study, 12 565 had breastfeeding data, 11 414 had complete attainment data, 4918 had non-missing breastfeeding and IQ data and 4152 were complete cases (individuals with breastfeeding, IQ and complete covariate information, but not necessarily linked attainment data) (Figure 1). Table 1 gives the numbers with and without linked attainment data according to completeness of ALSPAC data.

**Table 1.** Completeness of ALSPAC data according to availability of linked attainment data^a^

Complete data on:			Linked attainment data		Total
Covariates	Breastfeeding	IQ	Yes	No	
Yes	Yes	Yes	3605	547	4152
		No	4135	1087	5222
	No	Yes	32	6	38
		No	197	81	278
No	Yes	Yes	692	74	766
		No	1949	476	2425
	No	Yes	55	12	67
		No	749	278	1027
			11 414	2561	13975

^a^Among the 13 975 singletons and twins alive at one year.

Table 2 shows predictors of missing IQ among individuals with breastfeeding, complete covariate information and linked attainment data (*n* = 7740). Females were less likely to have missing IQ, as were firstborn children and those born to mothers who did not smoke during pregnancy, were older and had higher socioeconomic status (according to education and other factors). Increased duration of breastfeeding was also associated with lower odds of having missing IQ data. After mutually adjusting for all other factors, father’s education, family adversity index and ethnicity were not related to missing IQ (results not shown). Lower attainment was strongly related to having missing IQ data, even after adjusting for these factors.

**Table 2.** Predictors of missing IQ at age 15 years (*n* = 7740, individuals with breastfeeding data, complete covariate information plus linked attainment data)

Factor	Level	OR (95% CI)^a^	*P*-value
Five or more A–C grades at Key Stage 4	No	1.00	=0.05
	Yes	0.85 (0.73, 1.00)	
Key Stage 4 capped point score	(OR for each 10-point increase)	0.96 (0.95, 0.97)	<0.001
Sex	Male	1.00	<0.001
	Female	0.85 (0.78, 0.94)	
Mother’s education	O level or lower	1.00	<0.001
	A level	0.82 (0.73, 0.92)	
	Degree or higher	0.85 (0.71, 1.02)	
Duration of breastfeeding	Never / less than 1 month	1.00	<0.001
	1 to <3 months	0.75 (0.64, 0.87)	
	3 to <6 months	0.68 (0.59, 0.79)	
	6 months +	0.60 (0.53, 0.68)	
Mother’s age at birth	<20	1.00	<0.001
	20–24	0.72 (0.48, 1.06)	
	25–29	0.62 (0.42, 0.91)	
	30–34	0.50 (0.34, 0.75)	
	35+	0.45 (0.29, 0.68)	
Family occupational social class	I-IIIn	1.00	=0.16
	IIIm-V	1.11 (0.96, 1.28)	
Mother smoked during pregnancy	No	1.00	<0.001
	Yes	1.31 (1.15, 1.49)	
Parity	0	1.00	<0.001
	1	1.30 (1.17, 1.45)	
	2	1.60 (1.37, 1.87)	
	3+	1.77 (1.37, 2.37)	
Housing tenure in pregnancy	Mortgaged/owned	1.00	<0.001
	Private rented	1.63 (1.30, 2.06)	
	Council/HA rented	1.19 (0.98, 1.44)	
	Other	1.14 (0.84, 1.56)	

HA, housing association.

^a^Mutually adjusted for all covariates (factors not associated with missing IQ: father’s education and family adversity index—not shown).

Subjects whose parents were more highly educated were less likely to have linked attainment data, as were those with higher IQs; there was also evidence that females were slightly less likely to have attainment data (Table 3). After adjusting for these factors, none of the other variables was related to missing attainment (results not shown).

**Table 3.** Predictors of missing Key Stage 4 attainment data among complete cases (*n* = 4152)

Factor	Level	OR (95% CI)^a^	*P*-value
IQ	(OR for each 10-point increase)	1.21 (1.11, 1.31)	<0.001
Sex	Male	1.00	=0.17
	Female	0.88 (0.73, 1.06)	
Mother’s education	O level or lower	1.00	<0.001
	A level	1.38 (1.09, 1.76)	
	Degree or higher	1.83 (1.37, 2.44)	
Father’s education	O level or lower	1.00	<0.001
	A level	1.41 (1.11, 1.81)	
	Degree or higher	1.69 (1.28, 2.23)	

^a^Mutually adjusted for all covariates (factors not associated with missing attainment not shown).

**Figure 2.**
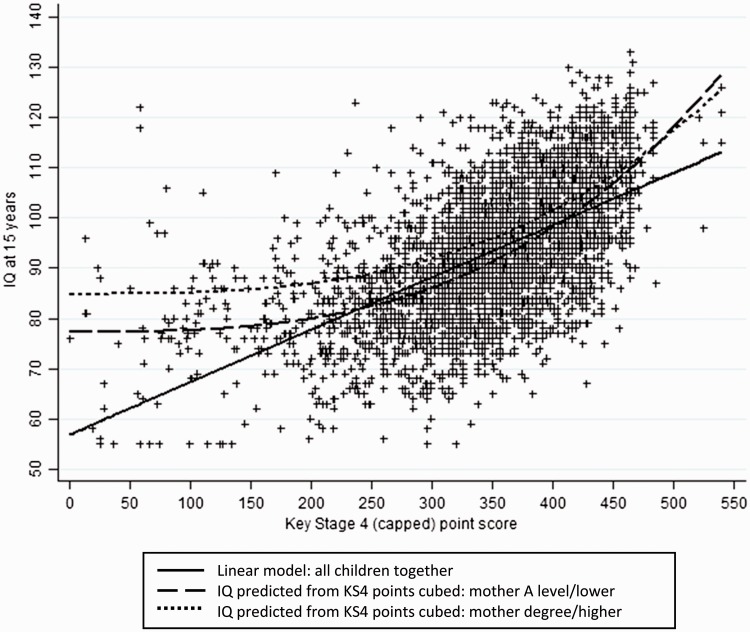
Relationship between Key Stage 4 (KS4) point score and IQ at 15 years. The y-axis starts at 50 to reflect the range of actual IQ scores (55–136).

We investigated the use of attainment score as a proxy for IQ (among the 4384 individuals with IQ and attainment). The best-fitting one-power fractional polynomial modelled IQ against attainment score cubed. Because the best-fitting two-power model (terms in attainment score squared and attainment score squared multiplied by the log of attainment) did not have a significantly better fit than the one-power model (change in deviance = 34380.1 -34376.1 = 4.0, *P* = 0.14) the one-power model was chosen. Cubed attainment score explained just under 40% of the variability in IQ (R^2^ = 0.39). There was evidence that this relationship differed by mother’s education: *P* (for interaction) = 0.1, after adjustment for all other covariates. Figure 2 shows the relationship between cubed attainment score and IQ separately by mother’s education; also shown is the linear model (all children together).

The mean (SD) IQ score among those with IQ data was 94.4 (13.1) points; among complete cases it was slightly higher: 95.3 (13.0). When IQ was imputed, the mean was 91.0 (13.1) when attainment was excluded and 89.4 (13.4) when it was included. Table 4 shows the predicted mean difference in IQ score for increasing duration of breastfeeding obtained using the five approaches. In all analyses, increased duration of breastfeeding was associated with higher mean IQ at 15—the estimated mean difference in IQ comparing those breastfed for at least 6 months with those never breastfed or breastfed for less than 1 month in the MI model using linked attainment data was 4.2 [95% confidence interval (CI) 3.4, 5.0). The two approaches incorporating attainment data gave similar results. Compared with these analyses, the analyses that did not take account of attainment resulted in slight underestimates of the effect of breastfeeding. As expected, there was more bias in the unadjusted estimates. The complete case analysis and MI including only baseline factors gave similar results. Often researchers compare the results obtained using these two approaches and, if similar, conclude that bias is unlikely. However, if the outcome variable is MNAR these methods may give similarly biased results.

**Table 4.** Relationship between duration of breastfeeding and IQ at 15

		Analysis approach				
		Excluding attainment data			Including attainment data	
Duration of breastfeeding		Complete case analysis (*n* = 4152)	Inverse probability of missingness weighting (*n* = 4152)	Multiple imputation (*n* = 13 975)	Inverse probability of missingness weighting (*n* = 3605)	Multiple imputation^b^ (*n* = 13 975)
Never / less than 1 month	Unadjusted difference in mean IQ (95% CI)	–—	–—	–—	–—	–—
1 to <3 months		1.9 (0.6, 3.2)	2.1 (0.7, 3.5)	2.7 (1.5, 3.9)	2.5 (0.9, 4.0)	3.2 (2.2, 4.3)
3 to <6 months		5.1 (4.0, 6.3)	5.6 (4.4, 6.8)	6.1 (5.1, 7.0)	5.5 (4.1, 6.8)	6.6 (5.6, 7.6)
6 months+		7.5 (6.6, 8.5)	8.0 (7.0, 9.0)	8.5 (7.6, 9.3)	8.2 (7.1, 9.3)	9.3 (8.5, 10.1)
Never / less than 1 month	Adjusted^a^ difference in mean IQ (95% CI)	–—	–—	–—	–—	–—
1 to <3 months		0.8 (-0.4, 2.0)	0.9 (-0.4, 2.2)	0.8 (-0.4, 2.0)	1.4 (-0.02, 2.9)	1.3 (0.3, 2.4)
3 to <6 months		2.6 (1.5, 3.7)	2.8 (1.7, 4.0)	2.6 (1.6, 3.6)	3.0 (1.7, 4.3)	3.2 (2.2, 4.2)
6 months+		3.5 (2.5, 4.4)	3.7 (2.7, 4.7)	3.3 (2.4, 4.2)	4.3 (3.2, 5.5)	4.2 (3.4, 5.0)

KS4, Key Stage 4.

^a^A djusted for sex, maternal and paternal education, occupational social class, parity, maternal age, ethnicity, family adversity index and housing tenure during pregnancy.

^b^IQ predicted from KS4 points cubed (best fitting fractional polynomial of degree 1), plus all other factors. Imputation model for IQ also included an interaction between KS4 points cubed and mother’s education.

The Hosmer–Lemeshow test did not indicate a poor fit when attainment data were included in the model predicting missing IQ (χ_8_^2^ = 5.9, *P* = 0.7); conversely, when attainment data were excluded, the model did not fit as well (χ_8_^2^ = 12.2, *P* = 0.1). When large weights were truncated in the IPMW, the estimated effect of breastfeeding was slightly reduced as the maximum value was decreased (Supplementary Table 1, available as Supplementary data at *IJE* online). Compared with the complete case analysis, MI incorporating attainment data was more efficient, reducing the standard errors for the breastfeeding estimates by 11–15% in the adjusted model.

When we deducted 10 points from the imputed IQs of those with missing attainment data, the crude estimates of the effect of breastfeeding were 3.3, 6.5 and 9.2 for the three breastfeeding groups, respectively; the adjusted estimates were 1.3, 3.1 and 4.2, respectively.

## Discussion

We have found that, conditional on parental education and other baseline socio-demographic factors, IQ measured at 15 years in ALSPAC appears to be MNAR; thus, the complete case analysis as well as the IPMW and MI without linked attainment data give biased estimates of the effect of breastfeeding. With the addition of attainment, a strong predictor of both IQ and missing IQ, it is more likely that IQ is MAR, although this assumption is not testable. In this situation, IPMW and MI would give unbiased estimates. Because of the relatively large amount of variation in IQ for a given level of attainment, it is possible that IQ remains MNAR, albeit to a lesser extent, even after including attainment; as such, some bias could remain. Our results also indicate that the positive effect of breastfeeding on IQ extends into adolescence, although we are cautious about making statements about the effect size since we have not set out to fully adjust for confounding.

One strength of this study is that we were able to take account a range of socio-demographic factors—predictors of non-response as well as potential confounders of the breastfeeding-IQ relationship. This was a large study and we were able to obtain linked attainment data for 82% of subjects. The main limitation is that attainment is not available for all children. There are several reasons for this. A certain proportion will be missing because the NPD only covers English schools and some will be missing because we did not have sufficient information to establish a link. However, more problematic is the fact that a large proportion of unlinked individuals would have been attending an independent school at the time of the linkage. It is possible that the relationship between IQ and attainment is different among those without attainment data. We have tried to capture this by including an interaction between mother’s education and attainment in the MI models. It is plausible that the relationship may be different again among children attending independent schools; our analyses would not capture this. However, our sensitivity analysis suggests that this is likely to have had little or no impact on the estimates of the effect of breastfeeding. Further, we would not expect there to be a strong impact because breastfeeding was not associated with missing attainment after taking account of parental education and other factors.

Other studies have used linked administrative data to quantify the bias due to missing observational data;^15–17^ however, these have generally been focused on bias in estimated rates, percentages or means rather than in exposure-outcome associations. Others^18^^,^^19^ have linked observational and administrative data to reduce bias, but used information on covariates collected as part of a survey to impute covariates (which are missing by design) in the administrative data. Yucel and Zaslavsky^20^ imputed treatment status, using linked survey and administrative data to correct for underreporting of cancer therapy in the administrative data. Faris *et al.*^21^ used administrative data to impute covariates missing in a study of heart disease and used logistic regression to look at associations between these covariates and mortality. The odds ratios obtained after imputing using only the study data were very similar after additionally including variables from the administrative data. However, they felt that their covariates were plausibly MAR (they did not use the administrative data to examine this). More recently, Hebert *et al*.^22^ carried out a simulation study using blood pressure data from medical records to impute follow-up blood pressure data missing in a clinical trial. They found that when the follow-up measurements were MNAR, MI incorporating the linked data reduced—but did not eliminate—bias in blood pressure measurements. They did not examine exposure-outcome associations.

In conclusion, our study indicates that incorporating linked attainment data into analyses can potentially reduce bias due to missing IQ in observational studies. Although the bias in this case appears to be relatively small, this may not always be the case. Being able to access—via linkage—a proxy for the outcome allows assessment of whether the probability of this outcome being missing depends on its unknown value and, when incorporated in analyses, makes an MAR assumption more plausible. Linkage to a proxy also allows more accurate prediction of the missing values. It is important to add that the utility of this will depend on what is being measured and the strength of association between the original outcome and its proxy. Further, it will depend on the coverage of the linked dataset and, in particular, on whether the coverage is independent of the outcome being studied. The advantages offered by linkage are of particular benefit when the outcome being studied is MNAR because in this situation the usual approaches to handling missing data will produce biased results.

## Supplementary Data

Supplementary data are available at *IJE* online.

## Funding

This work was supported by the UK Medical Research Council (MR/L012081/1). A.B. and A.D. are supported by the Wellcome Trust (WT086118/Z/08/Z). The UK Medical Research Council (MRC), the Wellcome Trust and the University of Bristol currently provide core funding for ALSPAC (WT092731). Data collection is funded from a wide range of sources.

## Supplementary Material

Supplementary Data
