# Supplementary material for: Using linked educational attainment data to reduce bias due to missing outcome data in estimates of the association between the duration of breastfeeding and IQ at 15 years
Source: Int J Epidemiol. 2015 Apr 8;44(3):937–45. doi: 10.1093/ije/dyv035 (PMC4521129; doi:10.1093/ije/dyv035)
Supplement: Supplementary Data [file supp_44_3_937__index.html]

Using linked educational attainment data to reduce bias due to missing outcome data in estimates of the association between the duration of breastfeeding and IQ at 15 years — Using linked educational attainment data to reduce bias due to missing outcome data in estimates of the association between the duration of breastfeeding and IQ at 15 years — Supplementary Data 

# Using linked educational attainment data to reduce bias due to missing outcome data in estimates of the association between the duration of breastfeeding and IQ at 15 years

## Supplementary Data

files

**Files in this Data Supplement:**

- Supplementary Data - docx file
