# Supplementary material for: Using linked educational attainment data to reduce bias due to missing outcome data in estimates of the association between the duration of breastfeeding and IQ at 15 years
Source: Int J Epidemiol. 2015 Apr 8;44(3):937–45. doi: 10.1093/ije/dyv035 (PMC4521129; doi:10.1093/ije/dyv035)
Supplement: Supplementary Data [file supp_dyv035_BFIQpaper_Supplementary_Table_1.docx]

Supplementary Table 1: Results for inverse probability weighting including linked attainment data, using different maximum values for truncating large weights

| Duration of  Breastfeeding |  | Actual weights used^2^  (n=3605) | Truncating large weights at: | | |
| --- | --- | --- | --- | --- | --- |
|  |  |  | 8  (n=3605) | 6  (n=3605) | 4  (n=3605) |
| Never / less than 1 month  1 to <3 months  3 to <6 months  6 months + | Unadjusted difference in mean IQ (95% CI) | ---  2.5 (0.9, 4.0)  5.5 (4.1, 6.8)  8.2 (7.1, 9.3) | ---  2.4 (0.9, 4.0)  5.4 (4.1, 6.8)  8.1 (7.0, 9.3) | ---  2.3 (0.8, 3.8)  5.3 (4.0, 6.6)  8.0 (6.9, 9.1) | ---  2.1 (0.7, 3.5)  5.0 (3.7, 6.3)  7.6 (6.6, 8.7) |
| Never / less than 1 month  1 to <3 months  3 to <6 months  6 months + | Adjusted^1^ difference in mean IQ (95% CI) | ---   - 1. (-0.02, 2.9)   3.0 (1.7, 4.3)  4.3 (3.2, 5.5) | ---  1.4 (-0.03, 2.9)  3.0 (1.7, 4.3)   - 1. (3.2, 5.5) | ---  1.3 (-0.1, 2.7)  2.9 (1.6, 4.2)  4.2 (3.1, 5.3) | ---  1.2 (-0.2, 2.6)  2.7 (1.5, 4.0)  4.0 (2.9, 5.1) |

1. Adjusted for sex, maternal and paternal education, occupational social class, parity, maternal age, ethnicity, family adversity index and housing tenure during pregnancy.
2. These results are the ones shown in Table 3.
